# Supplementary material for: Deacetylation as a receptor-regulated direct activation switch for pannexin channels
Source: Nat Commun. 2021 Jul 23;12:4482. doi: 10.1038/s41467-021-24825-y (PMC8302610; doi:10.1038/s41467-021-24825-y)
Supplement: Supplementary file 1 — Supplementary Information [file 41467_2021_24825_MOESM1_ESM.pdf]

Supplementary Information

Deacetylation as a receptor-regulated direct activation switch for pannexin channels

Yu-Hsin Chiu[1,2,3,*], Christopher B. Medina[4], Catherine A. Doyle[1], Ming Zhou[5], Adishesh K. Narahari[1], Joanna K. Sandilos[1], Elizabeth C. Gonye[1], Hong-Yu Gao[2], Shih Yi Guo[3], Mahmut Parlak[4], Ulrike M. Lorenz[4], Thomas P. Conrads[5], Bimal N. Desai[1], Kodi S. Ravichandran[4] and Douglas A. Bayliss[1,*]

1. *Department of Pharmacology, University of Virginia, Charlottesville, VA, USA*
2. *Institute of Biotechnology, National Tsing Hua University, Hsinchu, Taiwan*
3. *Department of Medical Science, National Tsing Hua University, Hsinchu, Taiwan*
4. *Department of Microbiology, Immunology & Cancer Biology, University of Virginia, Charlottesville, VA, USA*
5. *Inova Center for Personalized Health, Inova Schar Cancer Institute, Fairfax, VA, USA.*

* **Corresponding Authors**: dab3y@virginia.edu; yhchiu@life.nthu.edu.tw

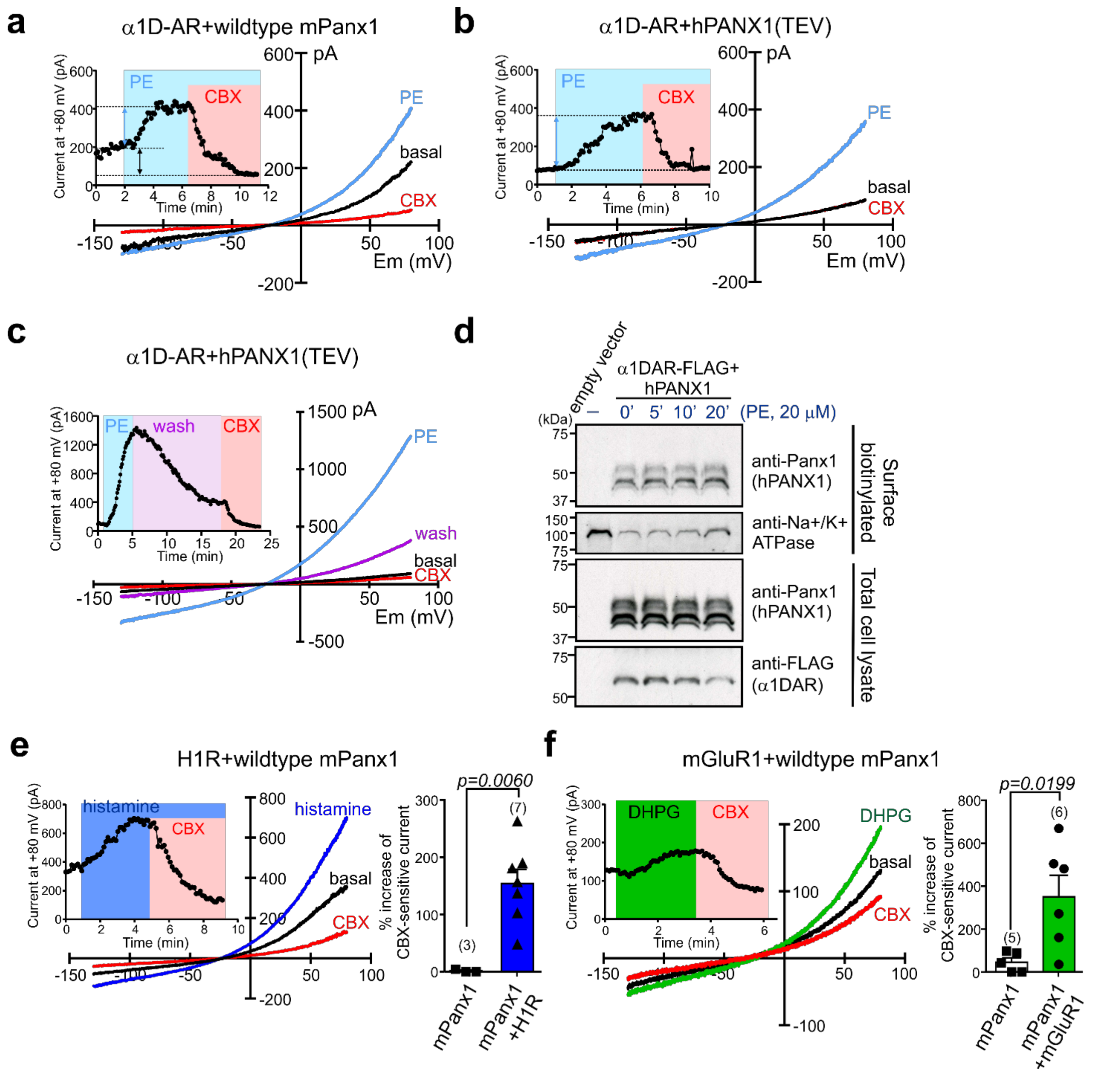

Supplementary Figure 1. Characteristics of Panx1 activation by Gαq-coupled receptors.
a,b) Exemplar records illustrating the effect of PE (20 μM) on whole cell currents in HEK293T cells expressing the α1D-AR and either wild type mPanx1 (**a**) or hPANX1(TEV) (**b**); as expected, Panx1 currents were inhibited by carbenoxolone (CBX, 50 μM). The *I*-V relationships were obtained from ramp voltage commands, and the time series from peak currents at +80 mV (*Insets*). The cell expressing mPanx1 displayed basal CBX-sensitive current (black double-headed arrow) that is not observed with hPANX1; for both, however, PE evoked an increase in CBX-sensitive current (cyan double-head arrow).

The hPANX1(TEV) construct substitutes a TEV protease site for the C-terminal caspase site, and cannot be activated by caspase-mediated cleavage[6]. **c)** The current induced by PE (20 μM) in HEK293T cells transfected with α1D-AR and hPANX1(TEV) was reversible, at least in part, after washout of the bath-applied agonist (n=9 cells examined over 3 independent experiments). **d)** In HEK293T cells transfected with α1D-AR and hPANX1, immunoblots (as a representative of n= 3 independent experiments) were performed with the indicated antibodies on total cell lysates or avidin-immunoprecipitates following cell surface biotinylation; PE (20 μM) had no effect on PANX1 channels at the cell surface, even after a 20-minute exposure. **e,f)** In HEK293T cells expressing mPanx1 together with the H1 histamine receptor (H1R) or the mGluR1 metabotropic glutamate receptor, treatment with the relevant agonist caused an increase in CBX-sensitive current. The *I*-V relationships were obtained from ramp voltage commands, and the time series from peak currents at +80 mV (*Insets*). The bar graphs present grouped data for CBX-sensitive current evoked by histamine (100 μM; **e**) or by DHPG (20 μM; **f**) from cells expressing mPanx1, with or without H1 (n=3 or 7 cells examined over 3 independent experiments) or mGluR1 (n=5 or 6 cells examined over 3 independent experiments) receptors. Two-tailed unpaired *t*-test (H1R: t=3.709, df=8; mGluR1: t=2.826, df=9). Summary data are presented throughout as mean ± s.e.m.

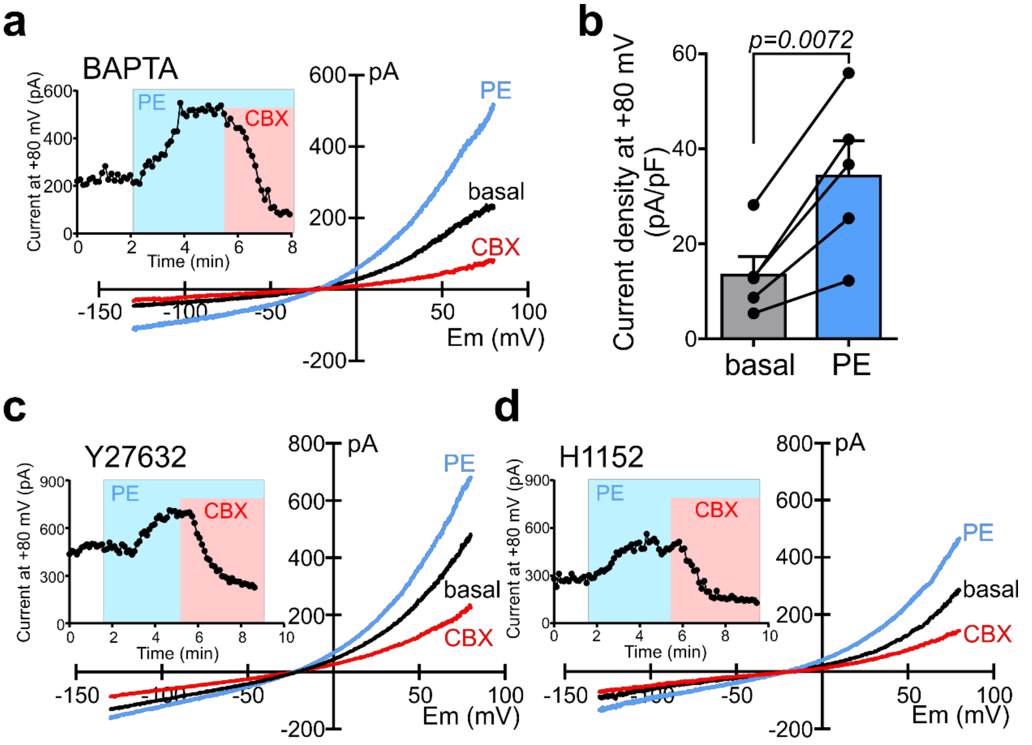

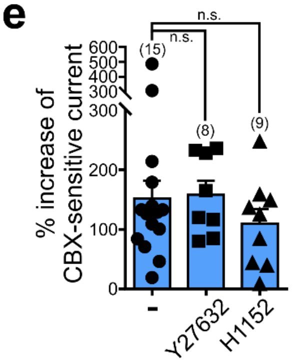

f

RhoA mutants	ROCK	mDia	Rhophilin	Citron	PKN	Kinectin
RhoA G14V	++	++	++	++	++	++
RhoA G14V/F39A	-	++	-	-	-	-
RhoA G14V/T37Y	-	-	-	-	-	-

Supplementary Figure 2. α1D receptor-mediated Panx1 activation does not require increased intracellular calcium and is independent of ROCK.

a,b) Effect of PE on whole cell currents in an α1D-AR/mPanx1-transfected HEK293T cell recorded with a pipette containing 14.3 mM BAPTA (pCa~8). **b)** Summary data (mean ± s.e.m) showing PE-activation of Panx1 currents from BAPTA-loaded cells (n=5 cells examined over 3 independent experiments). Two-tailed paired *t*-test (t=5.065, df=4). **c,d)** Effect of the ROCK inhibitors, Y27632 (10 µM, **c**) and H1152 (2 µM, **d**) on PE-activated whole cell currents in α1D-AR/mPanx1-transfected cells. **e)** Summary data (mean ± s.e.m) reveal that ROCK inhibitors had no effect on PE-activated Panx1 currents. n=15, 8, 9 cells, as indicated, examined over 5 independent experiments. n.s., not significant, by one-way ANOVA ($F_{2,29}$=0.7221, *p=0.4942*). **f)** Table showing effector activity for different constitutively active RhoA(G14V) mutant constructs (adapted from Tosello-Trampont et al.[23]).

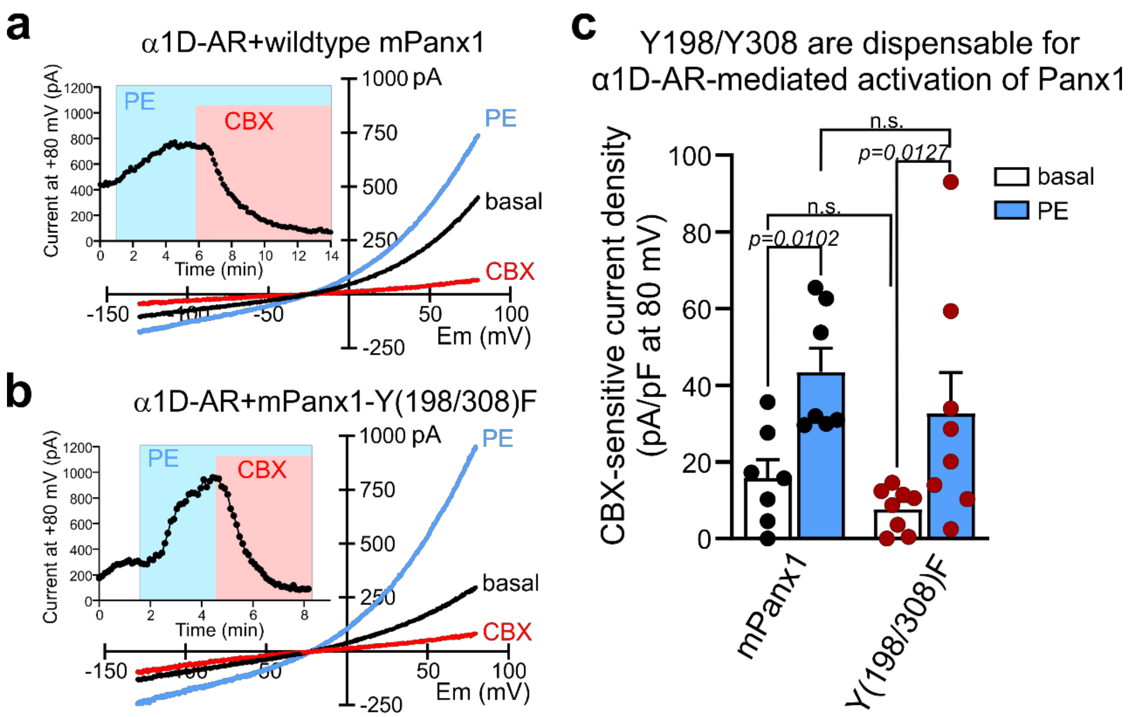

Supplementary Figure 3. Activation of Panx1 by α1D-ARs does not require tyrosine phosphorylation at Y198 or Y308.

a-b) Exemplar whole cell recordings showing PE-induced currents in HEK293T cells co-expressing α1D-ARs with either wild type mPanx1 (**a**) or a mutated mPanx1 in which Tyr-198 and Tyr-308 residues were substituted with phenylalanine and cannot be phosphorylated (**b**). **c**) Summary data (mean ± s.e.m) showing similar levels of PE-induced, CBX-sensitive current density in cells expressing wild type mPanx1 (n=7 cells examined over 3 independent experiments) and mPanx1(Y198/308)F (n=8 cells examined over 3 independent experiments); Two-way ANOVA ($F_{1,13}$=21.83, *p=0.0004*) with Sidak's multiple comparisons test (*p* values from comparisons are indicated; n.s, not significant).

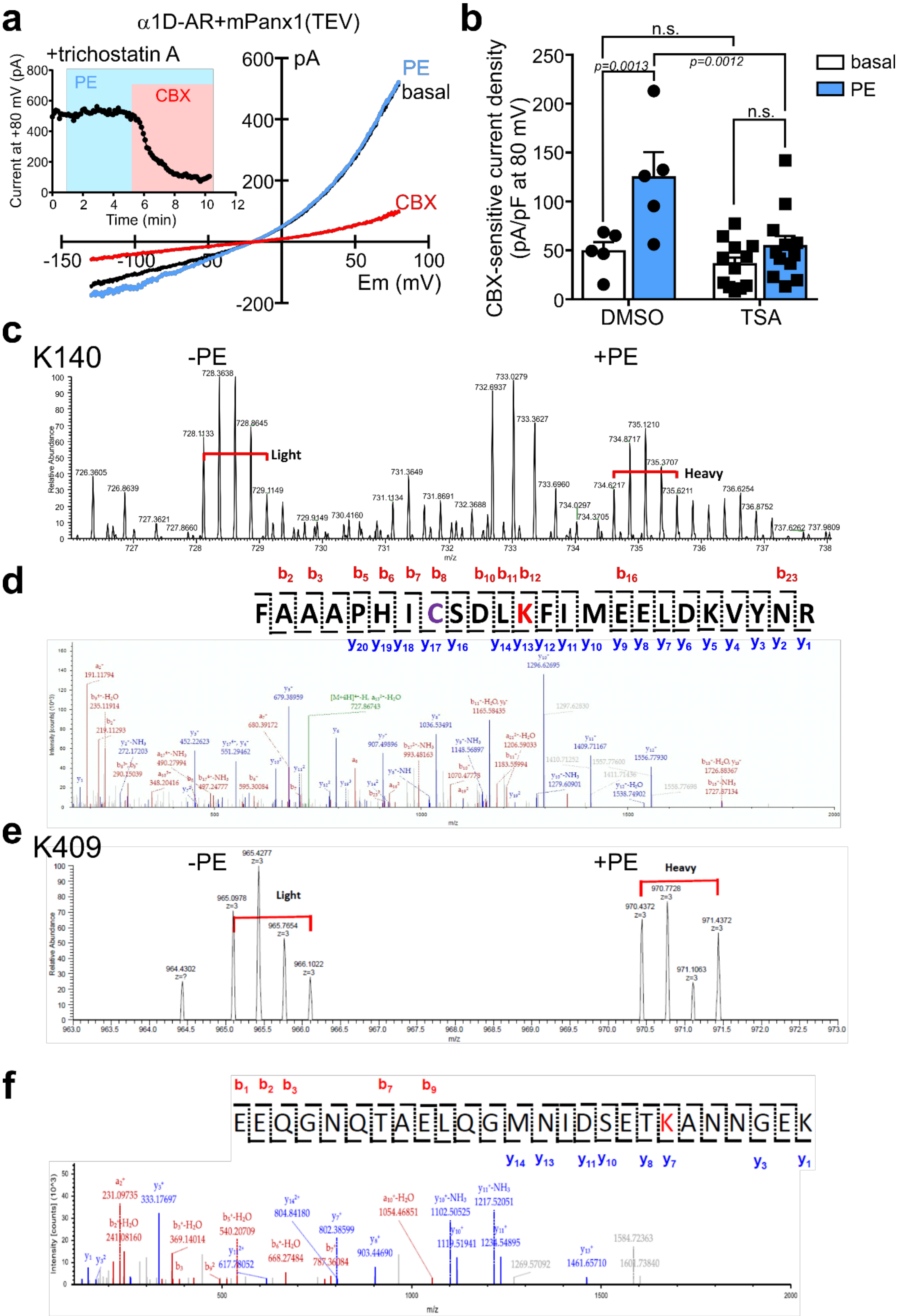
a
α1D-AR+mPanx1(TEV)
+trichostatin A
Current at +80 mV (pA)
PE
CBX
Time (min)
600 pA
PE
basal
CBX
Em (mV)
b
n.s.
p=0.0013
p=0.0012
n.s.
basal
PE
CBX-sensitive current density (pA/pF at 80 mV)
DMSO
TSA
c
K140
-PE
+PE
Light
Heavy
Relative Abundance
m/z
d
FAAAPHICSDLKFIMEELDKVYNR
e
K409
-PE
+PE
Light
Heavy
Relative Abundance
m/z
f
EEQGNQTAELQGMNIDSETKANNGEK
Intensity [counts] (10^3)
m/z

Supplementary Figure 4. α1D-AR-mediated activation of Panx1 involves protein deacetylation.

a) Exemplar records illustrating effect of PE in α1D-AR/mPanx1(TEV)-transfected HEK293T cell that was pre-treated with trichostatin A (TSA; 1 µM, ≥1h at 37°C), a broad-based HDAC inhibitor. **b**) Summary data (mean ± s.e.m) showing basal and PE-stimulated CBX-sensitive currents in vehicle (DMSO) and TSA-treated cells; TSA did not affect basal mPanx1 currents but inhibited PE-stimulated current. n=5 or 12 cells examined over 5 independent experiments. Two-way ANOVA ($F_{1,15}$=7.495, *p=0.0153*) with Bonferroni's multiple comparisons test (*p* values are indicated; n.s., not significant). **c-f**) MS1 and MS2 spectra of acetylated PANX1 peptides. Panx1 peptides were identified using Mascot (v1.36). The b (red) and y (blue) fragmentation ions are shown in the peptide sequences and the spectra. (**c**) MS1 spectrum of SILAC pair of PANX1 K140 acetylated, FAAAPHICSDLKFIMEELDKVYNR peptide. **d**) MS2 spectrum of acetylated peptide, FAAAPHICSDLKFIMEELDKVYNR, K12-Acetyl (42.01057 Da), C8-Carbamidomethyl (57.02146 Da), Charge: +4, Monoisotopic m/z: 728.11273 Da (-0.84 mmu/-1.15 ppm), MH+: 2909.42910 Da. **e**) MS1 spectrum of SILAC pair of PANX1 K409, acetylated, EEQGNQTAELQGMNIDSETKANNGEK peptide. **f**) MS2 spectrum of acetylated peptide, EEQGNQTAELQGMNIDSETKANNGEK, K409-Acetyl (42.01057 Da), M402-Oxidation (15.99492 Da), Charge: +3, Monoisotopic m/z: 965.09778 Da (+1.19 mmu/+1.23 ppm), MH+: 2893.27878 Da.

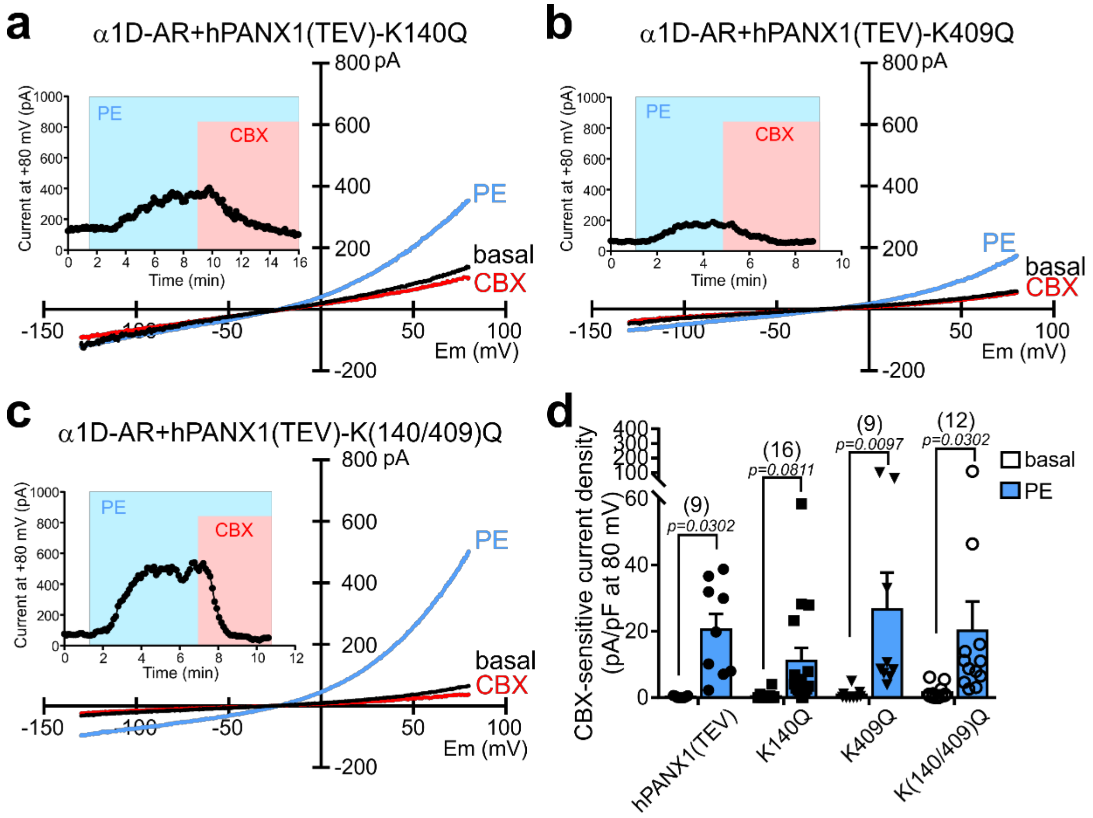

Supplementary Figure 5. Glutamine substitution at acetyl-lysine sites on PANX1 does not increase basal channel activity or occlude receptor activation.

a-c) Basal and PE-stimulated whole cell currents in α1D-AR-transfected HEK293T cells expressing the indicated Gln-substituted hPANX1 constructs, hPANX1(TEV)-K140Q, hPANX1(TEV)-K409Q or hPANX1(TEV)-K(140/409)Q). **d**) Summary data (mean ± s.e.m) reveal that basal current densities were not increased and PE-activation was unaffected by these single or double glutamine substitutions. n=9, 16, 9, or 12 cells examined over 5 independent experiments. Two-way ANOVA ($F_{1,42}$=26.92, $p<0.0001$) with Holm-Sidak's multiple comparisons test (*p* values are indicated).

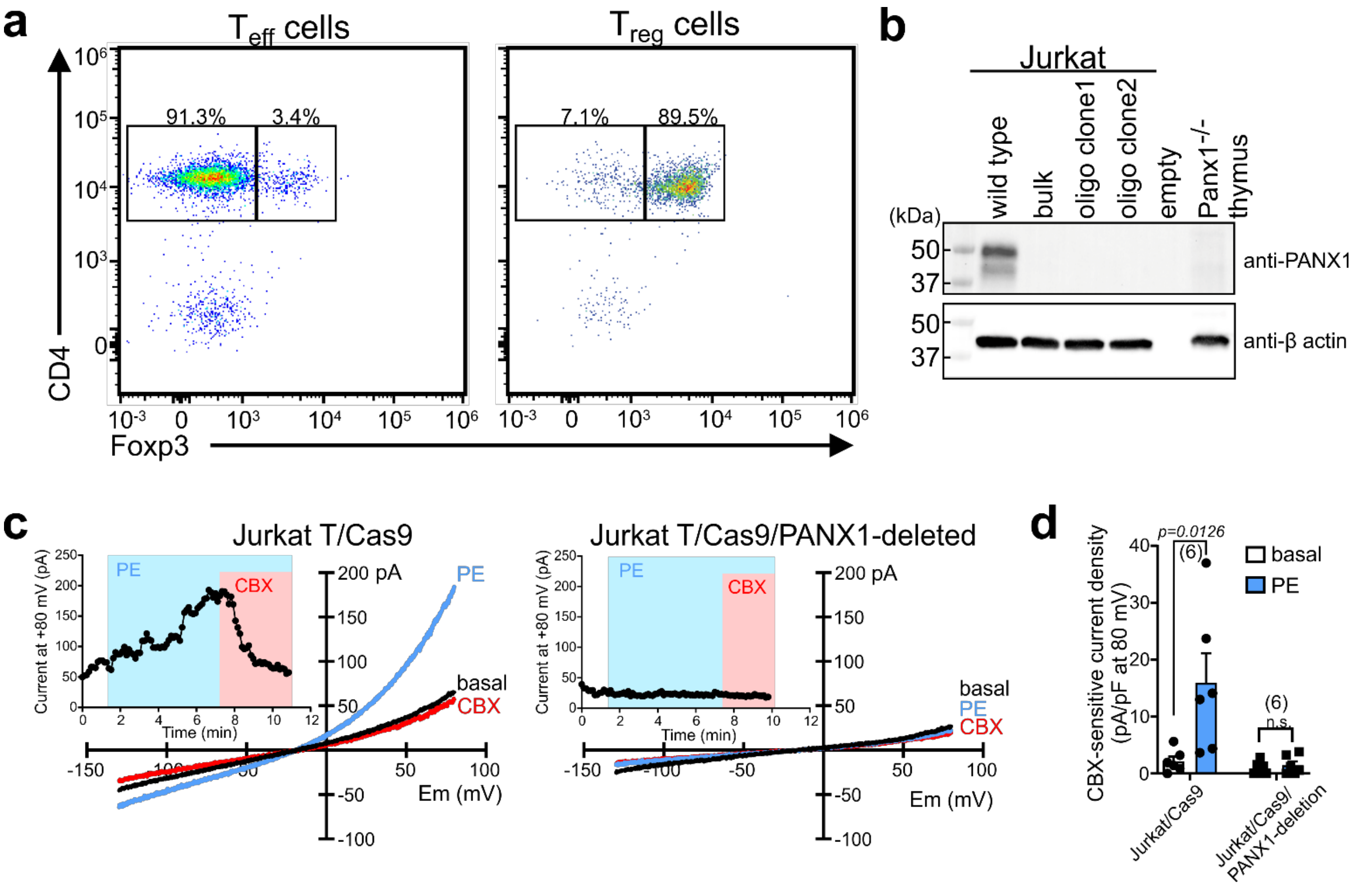

Supplementary Figure 6. α1-AR does not activate CBX-sensitive whole cell current in PANX1-deleted Jurkat cells.

a) Flow cytometry verification of isolated mouse CD4+ T cell populations. Representative flow cytometry data showing that the majority of isolated CD4+CD25- effector T cells (Teff, left) are CD4+Foxp3- (91.3%), while the majority of isolated CD4+CD25+ regulatory T cells (Treg, right) are CD4+Foxp3+ (89.5%). **b**) Western blots (representative from n=3 independent experiments) showing expression of PANX1 proteins in wild type Jurkat T cells, but not in PANX1-deleted Jurkat cells or the thymus of PANX1-null mice. *Bulk* indicates cells that were not clonally- selected, and *oligo* clones were derived from a few cells after serial dilutions. (**c**) Exemplar whole cell recordings from a parental Jurkat cell stably expressing Cas9 (*left*) and a PANX1-deleted Jurkat cell (*right*, data from oligo clone 2). (**d**) Grouped data (mean ± s.e.m) show that application of PE increased CBX-sensitive current density in parental Cas9-expressing Jurkat cells (n=6 cells examined over 3 independent experiments), whereas PE -induced current densities were not observed in any PANX1-deleted Jurkat cells (n=0/6 cells examined over 3 independent experiments). Analysis by two-way ANOVA $F_{1,10}$=5.479, *p=0.0413*, with Bonferroni's multiple comparisons test.

Supplementary Table 1: PANX1 peptides with acetylation at Lys140 or Lys409 identified by SILAC-LC-MS/MS analysis.

Biological Replicates	Peptide Group Modifications in Protein	Annotated Sequence	# PSMs	# Missed Cleavages	Abundance Ratio: (F1, Heavy) / (F1, Light)	Percolator PEP (by Search Engine): Mascot	Confidence (by Search Engine): Mascot	Abundance Ratio of Global PANX1 protein (F1, Heavy) / (F1, Light)	Normalized H/L Ratio: (Acetylated peptide/Global PANX1 peptides)
Replicate 1	1xAcetyl [K140]	[R]. FAAAPHICSDLK. [F]	2	0	0.706	0.003993	High	0.803	0.879
	1xAcetyl [K409]	[K].TPMSAEMREEQGNQTAELQGMNIDSETKANNGEK.[N]	2	2	0.692	0.000393	High	0.803	0.862
		[R].EEQGNQTAELQGMNIDSETKANNGEK.[N]	1	1	0.75	0.001828	High	0.803	0.934
		[R].EEQGNQTAELQGMNIDSETKANNGEK.[N]	1	1	0.764	0.0409	High	0.803	0.951
Replicate 2	1xAcetyl [K140]	[R].FAAAPHICSDLK.[F]	1	0	0.541	5.542E-07	High	0.59	0.917
		[R].FAAAPHICSDLK.[F]	1	0	0.588	4.407E-07	High	0.59	0.997
		[R].FAAAPHICSDLK.[F]	1	0	0.465	4.268E-07	High	0.596	0.780
		[R].FAAAPHICSDLK.[F]	1	0	0.484	0.00007544	High	0.596	0.812
	1xAcetyl [K409]	[K].TPMSAEMREEQGNQTAELQGMNIDSETKANNGEK.[N]	1	2	0.01	0.002023	High	0.59	0.017
Replicate 3	1xAcetyl [K140]	[R].FAAAPHICSDLKFIMEELDKVYNR.[A]	2	2	0.739	1.29E-08	High	0.553	1.336
		[R].FAAAPHICSDLK.[F]	2	0	0.517	0.05382	High	0.553	0.935
		[R].FAAAPHICSDLK.[F]	1	0	0.56	0.006496	High	0.551	1.016
		[R].FAAAPHICSDLK.[F]	1	0	0.01	8.09E-05	High	0.551	0.018
	1xAcetyl [K409]	[K].TPMSAEMREEQGNQTAELQGMNIDSETK.[A]	2	1	1.345	0.05382	High	0.551	2.441

Note: PE-treated samples: Heavy; unstimulated samples: Light. The acetylated lysine residues are indicated in red font in the peptide sequence. The peptides including C-terminally acetylated lysine (i.e., sites of trypsin/Lys-C digestion) were included in mass spectrometry identifications; the modified Lys residues were clearly recognized by their spectral characteristics and observed in all three analyses.

Supplementary Table 2: List of primers used for cloning and site-directed mutagenesis.

**Primer name**	**Primer sequence**
HDAC6 forward	5'-GACTCAGATCTATGACCTCAACCGGCCA-3'
HDAC6 reverse	5'-TTCGAAGCTTGTGTGGGTGGGGCATATC-3'
K140Q forward	5’-TTTGCTCAGACTTGCAGTTTATCATGGAAGAAC-3’
K140Q reverse	5’-GTTCTTCCATGATAAACTGCAAGTCTGAGCAAA-3’
K104R forward	5’-TTTGCTCAGACTTGAGGTTTATCATGGAAGAAC-3’
K104R reverse	5’-GTTCTTCCATGATAAACCTCAAGTCTGAGCAAA-3’
K409Q forward	5’-CATAGACAGTGAAACTCAAGCAAATAATGGAGAG-3’
K409Q reverse	5’-CTCTCCATTATTTGCTTGAGTTTCACTGTCTATG-3’
K409R forward	5’-CATAGACAGTGAAACTAGAGCAAATAATGGAGAG-3’
K409R reverse	5’-CTCTCCATTATTTGCTCTAGTTTCACTGTCTATG -3
