# Supplementary material for: Deacetylation as a receptor-regulated direct activation switch for pannexin channels
Source: Nat Commun. 2021 Jul 23;12:4482. doi: 10.1038/s41467-021-24825-y (PMC8302610; doi:10.1038/s41467-021-24825-y)
Supplement: Supplementary file 3 — Reporting Summary [file 41467_2021_24825_MOESM3_ESM.pdf]

nature research

Corresponding author(s): Douglas A. Bayliss; Yu-Hsin Chiu

Last updated by author(s): Jul 2, 2021

Reporting Summary

Nature Research wishes to improve the reproducibility of the work that we publish. This form provides structure for consistency and transparency in reporting. For further information on Nature Research policies, see Authors & Referees and the Editorial Policy Checklist.

Statistics

For all statistical analyses, confirm that the following items are present in the figure legend, table legend, main text, or Methods section.

n/a	Confirmed	
☐	☒	The exact sample size (*n*) for each experimental group/condition, given as a discrete number and unit of measurement
☐	☒	A statement on whether measurements were taken from distinct samples or whether the same sample was measured repeatedly
☐	☒	The statistical test(s) used AND whether they are one- or two-sided<br>*Only common tests should be described solely by name; describe more complex techniques in the Methods section.*
☐	☒	A description of all covariates tested
☐	☒	A description of any assumptions or corrections, such as tests of normality and adjustment for multiple comparisons
☐	☒	A full description of the statistical parameters including central tendency (e.g. means) or other basic estimates (e.g. regression coefficient) AND variation (e.g. standard deviation) or associated estimates of uncertainty (e.g. confidence intervals)
☐	☒	For null hypothesis testing, the test statistic (e.g. *F*, *t*, *r*) with confidence intervals, effect sizes, degrees of freedom and *P* value noted<br>*Give P values as exact values whenever suitable.*
☒	☐	For Bayesian analysis, information on the choice of priors and Markov chain Monte Carlo settings
☒	☐	For hierarchical and complex designs, identification of the appropriate level for tests and full reporting of outcomes
☒	☐	Estimates of effect sizes (e.g. Cohen's *d*, Pearson's *r*), indicating how they were calculated

Our web collection on statistics for biologists contains articles on many of the points above.

Software and code

Policy information about availability of computer code

Data collection	Axon pCLAMP10 (Molecular Devices); Softmax Pro 7 (Molecular Devices)
Data analysis	Axon pCLAMP10 (Molecular Devices); Softmax Pro 7 (Molecular Devices); Graphpad Prism ver.9; Proteome Discoverer (v.2.1.1.21, Thermo Fisher Scientific); Mascot (v. 2.5.1, Matrix Science Inc.); Percolator (v.3.5; http://percolator.ms/); FlowJo v.10.6.2 (FlowJo); ImageJ v.1.8 (NIH)

For manuscripts utilizing custom algorithms or software that are central to the research but not yet described in published literature, software must be made available to editors/reviewers. We strongly encourage code deposition in a community repository (e.g. GitHub). See the Nature Research guidelines for submitting code & software for further information.

Data

Policy information about availability of data

All manuscripts must include a data availability statement. This statement should provide the following information, where applicable:

- Accession codes, unique identifiers, or web links for publicly available datasets
- A list of figures that have associated raw data
- A description of any restrictions on data availability

The authors declare that all data supporting the findings of this study are available within the paper and its Supplementary Information/Source Data files. The raw mass spectrometry proteomics data have been deposited to the ProteomeXchange Consortium via the PRIDE partner repository with the dataset identifier PXD025912 [http://proteomecentral.proteomexchange.org/cgi/GetDataset?ID=PXD025912].

Field-specific reporting

Please select the one below that is the best fit for your research. If you are not sure, read the appropriate sections before making your selection.

☒ Life sciences ☐ Behavioural & social sciences ☐ Ecological, evolutionary & environmental sciences

For a reference copy of the document with all sections, see nature.com/documents/nr-reporting-summary-flat.pdf

Life sciences study design

All studies must disclose on these points even when the disclosure is negative.

Sample size: Sample sizes were not pre-determined; they were based on effect sizes and variability of data for each experiment.

Data exclusions: No data were excluded.

Replication: At least 3 biologically independent experiments were performed to confirm all results. For example, for recordings of whole cell currents or calcium transients, cells from each of the experimental conditions were always recorded from multiple independent preparations (transfections, isolations, etc.). Western blot analyses were performed at least 3 times each, and the replications are all successful.

Randomization: Interleaved recordings were performed from control and experimental cells in each recording session. Samples were allocated into different experimental groups based on varying treatments (transfection, chemical application, etc.).

Blinding: Analysis of mass spectrometry data was performed blinded to the experimental conditions. Investigators were not blinded for current or calcium recordings due to transfection conditions, in which various transfection reporters/indicators are required for conducting experiments.

Reporting for specific materials, systems and methods

We require information from authors about some types of materials, experimental systems and methods used in many studies. Here, indicate whether each material, system or method listed is relevant to your study. If you are not sure if a list item applies to your research, read the appropriate section before selecting a response.

Materials & experimental systems

n/a	Involved in the study	
☐	☒	Antibodies
☐	☒	Eukaryotic cell lines
☒	☐	Palaeontology
☐	☒	Animals and other organisms
☒	☐	Human research participants
☒	☐	Clinical data

Methods

n/a	Involved in the study	
☒	☐	ChIP-seq
☐	☒	Flow cytometry
☒	☐	MRI-based neuroimaging

Antibodies

Antibodies used: Primary antibodies used in this study include anti-Panx1 antibody (Cell Signaling Biotechnology; #91137), anti-FLAG M2 (Sigma-Aldrich, F3156), anti-Na+/K+-ATPase (Cell Signaling, #3010), anti-beta actin-Peroxidase (Sigma-Aldrich; #A3854) and anti-acetylated lysine antibody (Cell Signaling; #9441). Secondary antibodies used in this study include Amersham ECL Rabbit IgG, HRP-linked $F(ab')_2$ fragment (from donkey) (GE Healthcare; NA9340V; Lot# 9727551; 1:6000-1:10000) and Amersham ECL Mouse IgG, HRP-linked whole Ab (from sheep) anti-Mouse IgG (GE Healthcare; NA931V Lot# 15273046; 1:6000-1:10000).

Validation: Anti-FLAG M2 and anti-Na+/K+-ATPase were validated using immunobloting in our previous publications (PMID: 28134257). Recent publications from other groups also validate anti-acetylated lysine antibody (PMID: 30914652, 31015486), anti-beta actin-peroxidase (PMID: 31519886), and anti-Panx1 (PMID: 31118206).

Eukaryotic cell lines

Policy information about cell lines

Cell line source(s): HEK293T and Jurkat (E6.1) T cells were obtained from ATCC. Fq11 cells, embryonic fibroblasts derived from Gq/11 knockout mice, were kindly provided by Dr. Patricia Hinkle (University of Rochester, Rochester, NY), and Gq/11/12/13-deleted HEK293 cell was a generously gift from Dr. Asuka Inoue (Tohoku University, Miyagi, Japan).

Authentication: Fq11 and Gq/11/12/13-deleted HEK293 cells were functionally authenticated by measuring whole cell current or intracellular calcium following activation of Gq-coupled receptors, with or without reintroducing wild type Gq constructs (as shown in Fig. 1). HEK293T and Jurkat cells were not further authenticated after purchase.

Mycoplasma contamination	All cells were tested negative for mycoplasma contamination.
Commonly misidentified lines (See ICLAC register)	None of cell lines used in this study were registered as misidentified lines.

Animals and other organisms

Policy information about studies involving animals; ARRIVE guidelines recommended for reporting animal research

Laboratory animals	This study used wild type C57BL/6J mice of both sexes, aged 8 to 14 weeks.
Wild animals	This study does not involve wild animals.
Field-collected samples	This study does not involve samples collected from the field.
Ethics oversight	Use of mice for tissue harvest was approved by the Institutional Animal Care and Use Committee of the University of Virginia.

Note that full information on the approval of the study protocol must also be provided in the manuscript.

Flow Cytometry

Plots

Confirm that:

☒ The axis labels state the marker and fluorochrome used (e.g. CD4-FITC).

☒ The axis scales are clearly visible. Include numbers along axes only for bottom left plot of group (a 'group' is an analysis of identical markers).

☒ All plots are contour plots with outliers or pseudocolor plots.

☒ A numerical value for number of cells or percentage (with statistics) is provided.

Methodology

Sample preparation	Naïve CD4+ T cells were isolated from spleens of wildtype C57BL/6J mice (8~14 weeks; both sexes). In brief, dissected mouse spleens were gently ground through 70 µm cell strainer mesh (Falcon), and naïve CD4+/CD25+ (regulatory; Treg) or CD4+/CD25- (effector; Teff) T cells were isolated and collected using a CD4+CD25+ T cell isolation kit (Miltenyi Biotec; #130-091-041) and magnetic separation columns (Miltenyi Biotec, #130-042-201 and #130-042-401) according to manufacturer's protocol.
Instrument	Attune NxT (Invitrogen)
Software	FlowJo v.10
Cell population abundance	Purity of T cell populations were verified by using flow cytometry (CD3-PerCP-Cy5.5 1:100, eBioscience, #45-0031-80; CD4-Pacific Blue 1:100, eBioscience, #57-0042-82; and Foxp3-APC 1:100, eBioscience, #17-5773-80). Isolated CD4+ T cells were resuspended in RPMI1640 containing 10% FBS, 1% PSQ, 1% sodium pyruvate, 1% HEPES, and 1% non-essential amino acids, and plated on poly-L-lysine coated coverslips >1 h before recording.
Gating strategy	Standard lymphocyte gates were applied using FSCAxSSCA, following by doublet exclusion using FSCHxWand SSC-HxW. The T cell population was initially gated using CD3. T-effector cells and T-regulatory cells were gated based on CD4 and Foxp3, with T-effector cells being CD4+Foxp3- and T-regulatory cells being CD4+Foxp3+.

☒ Tick this box to confirm that a figure exemplifying the gating strategy is provided in the Supplementary Information.
